# Verification of the Laser Powder Bed Fusion Performance of 2024 Aluminum Alloys Modified Using Nano-LaB_6_

**DOI:** 10.3390/ma17133367

**Published:** 2024-07-08

**Authors:** Zeyu Yao, Ziwen Xie

**Affiliations:** 1 School of Design, Jiangnan University, Wuxi 214126, China; 2 School of Mechanical Engineering, Jiangnan University, Wuxi 214126, China; 6200810105@stu.jiangnan.edu.cn

**Keywords:** laser powder bed fusion, LaB_6_ nanoparticles, A2024 matrix composites, aerospace seats, mechanical properties

## Abstract

The application of 2024 aluminum alloy (comprising aluminum, copper, and magnesium) in the aerospace industry is extensive, particularly in the manufacture of seats. However, this alloy faces challenges during laser powder bed fusion (PBF-LB/M) processing, which often leads to solidification and cracking issues. To address these challenges, LaB_6_ nanoparticles have been investigated as potential grain refiners. This study systematically examined the impact of adding different amounts of LaB_6_ nanoparticles (ranging from 0.0 to 1.0 wt.%) on the microstructure, phase composition, grain size, and mechanical properties of the composite material. The results demonstrate that the addition of 0.5 wt.% LaB_6_ significantly reduces the average grain size from 10.3 μm to 9 μm, leading to a significant grain refinement effect. Furthermore, the tensile strength and fracture strain of the LaB_6_-modified A2024 alloy reach 251 ± 2 MPa and 1.58 ± 0.12%, respectively. These findings indicate that the addition of appropriate amounts of LaB_6_ nanoparticles can effectively refine the grains of 2024 aluminum alloy, thereby enhancing its mechanical properties. This discovery provides important support for the broader application of 2024 aluminum alloy in the aerospace industry and other high-performance fields.

## 1. Introduction

Laser additive manufacturing technology, specifically laser powder bed fusion (PBF-LB/M), is highly regarded in various technical fields and has found applications in the aerospace, biomedical, automotive, and other industries [1,2]. PBF-LB/M technology utilizes a computer-aided design model to efficiently melt successive layers of powdered material with a high-energy laser beam. The molten metals then solidify to create manufacturing components. In comparison to traditional manufacturing methods, PBF-LB/M technology offers advantages such as near-net forming and superior material utilization, allowing for the production of high-density, intricate, and irregularly shaped parts [3,4].

Research indicates that forged aluminum alloys, particularly the 2xxx, 6xxx, and 7xxx series, demonstrate superior strength compared to cast aluminum–silicon alloys [5]. A2024 aluminum alloy primarily consists of copper (Cu: 3.8–4.9%), magnesium (Mg: 1.2–1.8%), chromium (Cr: 0.10%), silicon (Si: 0.50%), zinc (Zn: 0.25%), and manganese (Mn: 0.30–1.0%), and is renowned for its high strength and the ability to be strengthened through heat treatment [6]. With a favorable strength–density ratio, ductility, and resistance to fatigue fracture under alternating stress, it has become a preferred material for demanding applications where performance is crucial. It has progressively established itself as the primary material for critical aerospace components, including aviation seats and bolts [7,8,9]. Nevertheless, the processability of A2024 aluminum alloy using PBF-LB/M technology is considerably limited. This constraint arises from a combination of factors: subpar fluidity; the relatively high degree of the effective reflection of light shining on its surface; and the high heat conduction efficiency of the A2024 aluminum alloy powder, which significantly impairs processing quality [10,11]. Additionally, the alloy’s high thermal expansion coefficient and broad solidification range render the forming process susceptible to freezing and cracking [5,12].

The processing of PBF-LB/M aluminum alloys has been extensively studied to enhance processability, address crack formation challenges, and maintain exceptional mechanical properties. Various strategies have been proposed, including adjusting laser processing parameters such as power, scanning speed, and spacing to alter solidification behavior and minimize crack formation. Despite these efforts, the complete elimination of cracks remains challenging [13,14,15]. In addition, the PBF-LB/M preparation of crack-free aluminum alloys can be achieved by refining equipment-related parameters, including pulsed laser [16], auxiliary heat source [17], and substrate preheating temperature [18]. The relatively high cost and low efficiency of aluminum alloys pose significant challenges to their widespread application and manufacturing.

Taking into account the aforementioned context, the research on eliminating cracks in PBF-LB/M aluminum alloys has gradually shifted from process optimization to adjusting the material’s composition. Specifically, the addition of effective heterogeneous nucleating agents, grain-refining particles, and other secondary nanoparticles has emerged as a crucial approach to mitigating solidification cracks in high-strength aluminum alloys suitable for PBF-LB/M [19,20]. Several secondary nanoparticles, including TiB_2_ [21,22], Si_3_N_4_ [23], ZrH_2_ [24,25], and SiC [26], have demonstrated beneficial effects on the PBF-LB/M process for aluminum alloys. Recent scientific reports have suggested that hexaborides, such as CeB_6_, LaB_6_, CaB_6_, SrB_6_, and NdB_6_, possess the capability to act as efficient grain refiners in aluminum alloys manufactured using laser powder bed fusion (PBF-LB/M). For example, considering the effect of CaB_6_ nanoparticles on LPBF treatment of 2024 aluminum alloy, the research results show that CaB_6_ significantly improves the processing performance of the alloy, almost completely eliminating cracks and pores, while inducing the transformation from columnar to equiaxed grains, significantly refining grain size, and significantly improving the mechanical properties of the alloy [27]. This capability arises from the nanoparticles’ cubic crystal structure and lattice parameters, which closely resemble those found in aluminum solid solutions. The integration of these hexaborides during the PBF-LB/M process is anticipated to enhance the mechanical properties and performance of aluminum alloys by facilitating grain refinement [28]. However, numerous studies have successfully produced numerous high-performance aluminum alloys by incorporating these nanoparticles [27,29,30,31,32]. To the best of the authors’ knowledge, there remain few systematically investigated LaB_6_/A2024 alloys prepared by PBF-LB/M.

The addition of secondary nanoparticles has a dual effect on the material. It not only refines the grain structure, thus mitigating solidification cracks, but also significantly alters mechanical properties through various strengthening mechanisms. These mechanisms encompass the strengthening of metal grain boundaries, grains themselves, and dislocation density. Unfortunately, enhancing the strength of aluminum alloys often comes at the cost of reduced ductility, and this reduction becomes more pronounced as the size, agglomeration, and content of the reinforced phase increase, resulting in stress concentration [33]. In summary, by carefully controlling the phase content of the reinforcing metal within a suitable range, the strength–ductility ratio of the metal alloy can be optimized. However, the excessive addition of nanoparticles can significantly compromise ductility [19,34,35]. Therefore, optimizing the quantity of the reinforcement phase added is paramount for producing high-quality PBF-LB/M components.

Hence, in addition to the original A2024 aluminum alloy powder, an LaB_6_/A2024 powder mixture with varying reinforcement phase contents (i.e., 0, 0.25, 0.5, 0.75, and 1.0 wt.%) was prepared. The PBF-LB/M process was employed to form the components, followed by a comprehensive analysis of their microstructure, phase composition, and mechanical properties.

## 2. Experimental Procedures

### 2.1. Materials and Powder Preparation

**Figure 1:**
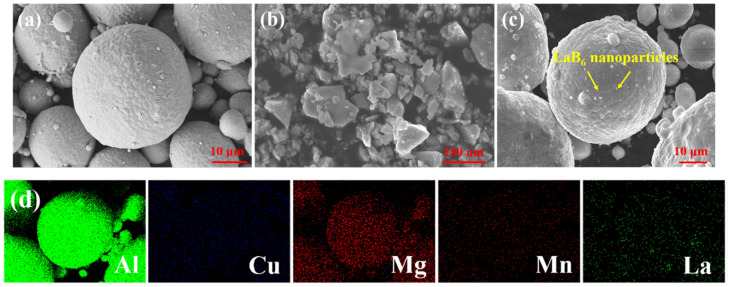
The morphology of feedstock powder: (**a**) the micrograph of A2024 powder, (**b**) the micrograph of LaB_6_ powder, (**c**) the micrograph of LaB_6_/A2024 mixed powder, and (**d**) the corresponding EDS maps of (**c**).

In this study, the raw material for the A2024 aluminum alloy powder was prepared using the gas atomization method. Table 1 outlines the chemical components of this powder, which exhibit a regular spherical shape and a typical unimodal particle size distribution, as shown in Figure 1a.

**Figure 2:**
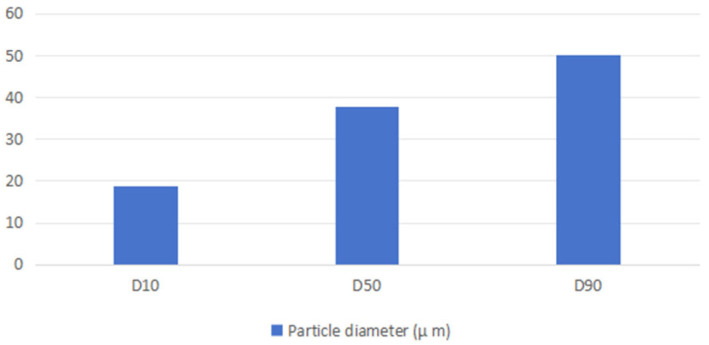
Particle size distribution histogram (μm).

The measured values for D10, D50, and D90 are 18.84 μm, 37.62 μm, and 50.07 μm, respectively, as shown in Figure 2

To enhance the performance of the alloy, scientifically graded LaB_6_ powders with particle sizes close to 50 nm and relatively irregular morphology were added. As shown in Figure 1b, these powders were mechanically mixed with the A2024 powder in an argon environment using a ball mill. The grinding conditions were optimized at a speed of 300 rpm, a duration of 160 min, and a ball–material ratio of 2:1. The LaB_6_ powder was added in varying amounts: 0.25, 0.5, 0.75, and 1.0 weight%. This approach was used to explore the influence of reinforcement phase contents on the microstructure, phase composition, and mechanical properties of the resulting materials processed via PBF-LB/M.

For the purposes of simplification, the printed LaB_6_/A2024 samples were designated as S_0_, S_0.2_, S_0.5_, S_0.75_, and S_1_, corresponding to the varying reinforcement phase contents of the LaB_6_ powder. Figure 1c demonstrates that the microstructure of the LaB_6_/A2024 mixed powder remains intact after ball milling, indicating no significant agglomeration or particle growth. Prior to the laser powder bed fusion (PBF-LB/M) process, all raw material powders were vacuum-dried at 100 °C for 8 h to eliminate any moisture content and ensure consistent fusion during the printing process. The corresponding EDS plot in Figure 1d further indicates the successful mixing of the sample.

### 2.2. Laser Powder Bed Fusion Processing

In this study, the required samples were manufactured using laser powder bed fusion (PBF-LB/M) technology on an XDM 120 machine equipped with a Yb 5500 W IPG fiber laser. The A2024 platform was selected as the building base for all samples, and the substrate was preheated to 200 °C to ensure optimal adhesion and minimize thermal stress during the printing process. To prevent oxidation and maintain the integrity of the printed parts, stringent measures were implemented to limit the oxygen content in the forming chamber to below 50 ppm throughout the PBF-LB/M process. These measures included the use of inert gases and a closed-loop oxygen control system to ensure high-quality printed samples.

**Figure 3:**
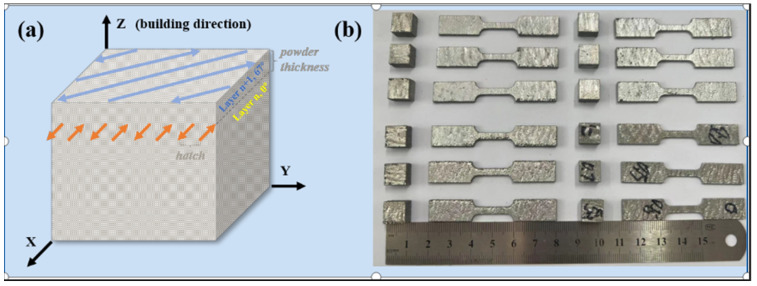
(**a**) Scanning strategy between consecutive layers and (**b**) tensile specimen details.

After careful optimization, the laser processing parameters were determined as follows: a laser power of 300 W, a scanning speed of 1300 mm/s, a pattern spacing of 120 μm, and a layer thickness of 30 μm. Additionally, a scanning rotation angle of 67° was employed between each layer to enhance mechanical performance and minimize anisotropy. These parameters were chosen to optimize the fusion quality, material distribution, and overall mechanical properties of the printed samples. Figure 3a actually depicts the scanning strategy employed during the PBF-LB/M process, illustrating the application of the optimized laser processing parameters.

To explore the material’s characteristics, we printed multiple cubes (10 × 10 × 5 mm^3^) and tensile bars following the ASTM E8 standard [36]. The tensile bars had a gauge length of 19.6 mm, a width of 5 mm, and a thickness of 2 mm, as illustrated in Figure 1b.

To ensure the reproducibility and consistency of the experimental results, three sets of replicate samples were fabricated under identical conditions.

### 2.3. Microstructural Characterization

The microstructure was analyzed using standardized techniques, which involved chemical etching at room temperature. The etching solution comprised 95 mL H_2_O, 2.5 mL HNO_3_, 1.5 mL HCl, and 1 mL HF per 100 mL of the solution. Microstructure characterization was conducted using a scanning electron microscope (SEM, FEI Nova Nano SEM 450, Hillsboro, OR, USA) equipped with an energy dispersive spectroscopy (EDS, Oxford X-Max 50, Shanghai, China) system. The phase identification of feedstock powders and specimens was performed using X-ray diffraction (XRD-D8 Advance, Beijing, China) with Cu-Kα radiation at 40 kV and 30 mA, employing a speed of 2°/min and scanning angle range of 20–90°. The grain size, morphology, and texture of each as-built sample were assessed through electron backscatter diffraction (EBSD). Furthermore, transmission electron microscopy (TEM; JEM-2100F, Changsha, China) was employed for detailed phase observation.

### 2.4. Mechanical Testing

The microhardness of the sample was evaluated on the polished XY plane using a Vickers hardness tester (HVS-1000ZCM-XY, Wuxi, China). A 200 g load was applied for 15 s, and ten indentations were randomly made on each sample. The average microhardness and standard deviation were then calculated. Room temperature tensile tests were carried out using an electronic universal testing machine (WDW-100KN, provided by Jinan Sida Testing Technology Co., Ltd., Wuxi, China) with an extensometer. The tensile rate was set at 0.5 mm/min, and three specimens from each sample group were tested, with the results averaged to determine a mean value.

## 3. Results

### 3.1. Phase Analysis

**Figure 4:**
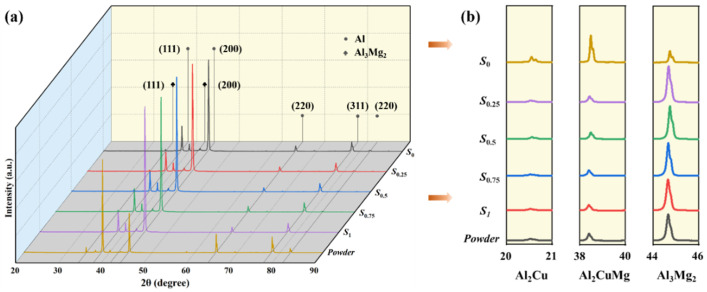
X-ray diffraction patterns of A2024 powder and constructed PBF-LB/M samples: (**a**) complete XRD patterns within the range of 20~90°; and (**b**) the amplified area of the pattern, representing low intensities.

The XRD pattern in Figure 4 displays the phase analysis of the powder and an XY cross-section of the PBF-LB/M samples.

It is observed that the peak intensities of some phases, such as α-Al, in the PBF-LB/M samples are generally lower compared to the initial powder, while other phases, like Al_2_CuMg, may exhibit comparable or even higher peak intensities. The PBF-LB/M-formed alloys primarily comprised face-centered cubic (fcc) α-Al with main orientations at (111), (200), (220), and (311). LaB_6_ peaks are not detected at the current XRD resolution due to the low addition amounts. Notably, the relative intensity of the α-Al Bragg’s peak remains consistent with respect to the increasing LaB_6_ amounts and no new phase is observed, indicating the high stability of the LaB_6_ nanoparticles. Apart from α-Al, which is the main phase, the intermetallic phases of Al_2_Cu, Al_2_CuMg, and Al_3_Mg_2_ are also present.

### 3.2. Microstructure Analysis

**Figure 5:**
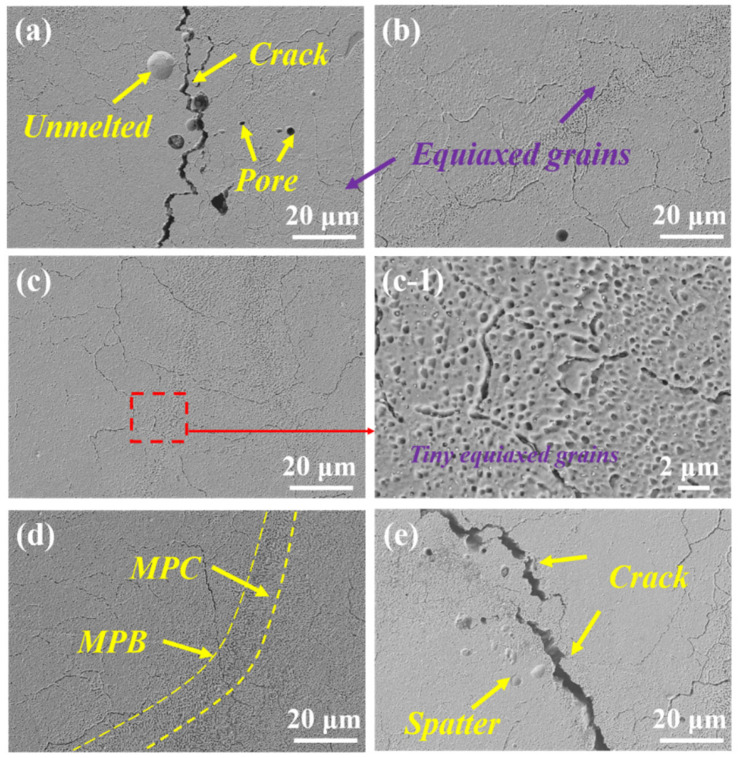
SEM morphology of the XY section microstructure of PBF-LB/M-formed LaB_6_/A2024 samples: (**a**) S_0_, (**b**) S_0.2_, (**c**) S_0.5_, (**d**) S_0.75_, (**e**) S_1_, and (**c-1**) partial magnification of (**c**).

Figure 5 presents the SEM morphology of the microstructure of the LaB_6_/A2024 alloy fabricated via PBF-LB/M. For sample preparation, the LPBF-formed samples were polished using standard metallographic techniques and etched with Keller’s reagent (HF:HCl:HNO_3_:H_2_O = 1:1.5:2.5:95) to reveal the microstructure.

It is evident that the addition of the heterogeneous nucleating agent LaB_6_ effectively mitigates crack defects in the PBF-LB/M-formed A2024. As shown in Figure 5a, the high thermal sensitivity of A2024 leads to severe cracking during rapid solidification. However, in the S1 sample, the cracks are mainly attributed to the agglomeration caused by the high content present in the reinforcement phase, which intensifies the stress concentration during the forming process. In the S_0.25_, S_0.5_, and S_0.75_ samples, the crack’s density significantly decreases. By performing comparisons in Figure 5, it can be observed that the pores in S0 are more obvious, with a significant reduction in pores at S_0.25_, S_0.5_, and S_0.75_. Based on experimental observations, porosity seems to have decreased and a more significant change was observed in S_0.5_ porosity. At this point, the second phases, LaB_6_ and α-, were observed. There are small differences in the atomic arrangement between different crystal structures in Al solid solutions, which helps to form coherent or semi-coherent interfaces. This results in α-. The nucleation barrier of the Al phase is relatively low, resulting in small equiaxed grains. α-Al grains are more adaptable to strain than hard, coarse columnar grains [37]. Moreover, the increase in the total grain boundary area per unit volume of metal reduces the thickness of its residual liquid film, preventing the initiation and propagation of cracks.

Figure 5 illustrates that both the A2024 aluminum alloy and its composites exhibit a typical equiaxed grain structure. In comparison to traditional casting or forging processes, the PBF-LB/M samples produce smaller equiaxed grain. While all samples display an interconnected grain boundary network, sample S_0_ without LaB_6_ nanoparticles not only has coarser grains but also exhibits unclear grain boundary regions. With the addition of LaB_6_, significant grain refinement and clear grain boundaries can be observed in S_0.25_ and S_0.5_ samples. The characteristics of the gray cells in the microstructure of PBF-LB/M samples all represent supersaturated primary α-Al. A partial magnification of Figure 5c-1 reveals that precipitates gather near grain boundaries, and the grain consists of numerous cellular dendrites, with dendrite arm spacing measuring 1~2 μm. It is worth noting that due to the morphological changes in the second phase of the melt pool, a flat melting boundary area can be found on the surface of the sample, and there are large dendrites distributed in the interface area. This can be attributed to the different cooling rates at different positions within the PBF-LB/M melt pool [38], as shown in Figure 5d.

### 3.3. Grain Morphology Evolution

**Figure 6:**
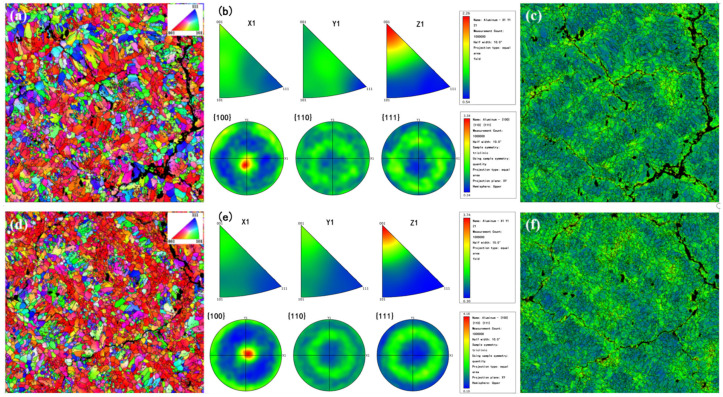
Horizontal EBSD inverse pole plot (EBSD-IPF): pole plots of (**a**–**c**) S_0_, (**d**–**f**) S_0.5_, and KAM. Observe the crystal orientation along the top surface (XY plane) and the IPF color of the sample, including the code representing the grain orientation. The KAM mapping is retrieved in degrees from the EBSD-IPF mapping in (**a**,**d**).

Figure 6 illustrates the EBSD-IPF diagrams along with their corresponding pole diagrams for all PBF-LB/M-formed alloys at XY cross-sections.

**Figure 7:**
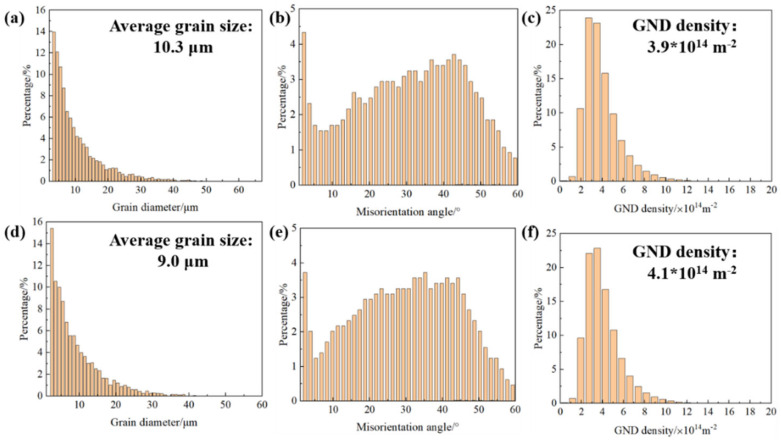
Average grain size of the PBF-LB/M-formed alloys. (**a**) S_0_ and (**b**) S_0.5_; misorientation angle of (**d**) S_0_ and (**e**) S_0.5_; and GND density of (**c**) S_0_ and (**f**) S_0.5_.

The grain size distribution, average grain size, dislocation angle, and GND density of all samples were derived from the EBSD-IPF diagram, as presented in Figure 7.

Notably, the S_0_ samples exhibit pronounced microstructural heterogeneity, characterized by a mixture of course-length columnar crystals and equiaxed crystals. While the proportion of equiaxed grains is relatively small, they exhibit random orientations. However, as opposed to the distribution of equiaxed grains, the color of columnar crystals in the sample is predominantly red, indicating that the crystal orientation of these grains is mainly (001), with the fiber texture being <001>. The corresponding pole figure (PF) indicates a maximum texture index of 3.39. Numerous intergranular solidification cracks are distributed on the samples’ surface, penetrating through the entire columnar grain, with the widest crack measuring approximately 14.1 μm, consistent with previous studies on the solidification crack behavior of PBF-LB/M-formed alloys [28,39].

LaB_6_ particles refined the grain sizes and caused a columnar to equiaxed (CET) structural transformation. By introducing 0.5 wt.% LaB_6_, the average grain size was reduced by 12.62% compared to that of the S_0_ alloy, decreasing it from 10.3 μm to a smaller size. This reduction in grain size increased the number of grain boundaries, promoting a more uniform distribution of residual stress at these boundaries. Consequently, the material’s crack sensitivity was reduced. Furthermore, a closer examination of the pole figure reveals a slight enhancement in the texture strength of S_0.5_ with increasing LaB_6_ content.

Figure 6c,f depict the KAM distribution of the two alloys. Near the grain boundary, the orientation deviation is approximately 2°, while within the grain, it is around 0°. Higher KAM values and nuclei with higher KAM values cluster near the solidification crack of the A2024 alloy. The required geometric dislocation density distribution diagram (GND) is shown in Figure 6c,f, and it reveals that the average GND density increases from 3.9 × 10^14^ m^−2^ to 4.1 × 10^14^ m^−2^ after adding the enhanced phase. This indicates that the addition of a reinforcing phase increases the dislocation density in the matrix, thereby enhancing the effect of dislocation reinforcement and helping to improve strength.

### 3.4. Mechanical Properties Evolution

**Figure 8:**
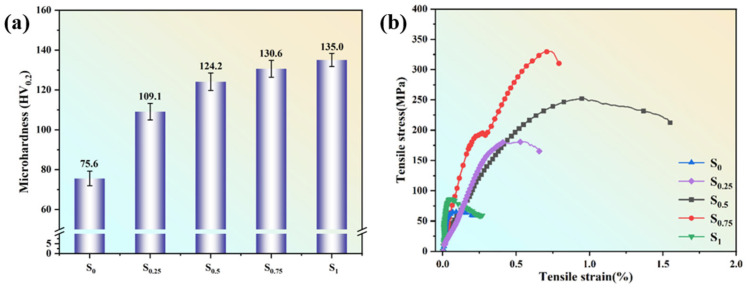
The mechanical properties of LaB_6_/A2024 samples prepared by LPFB. (**a**) Microhardness and (**b**) tensile performance.

From Figure 8a, it can be seen that the hardness of the sample increases with an increase in LaB_6_ content.

The hardness values of S_0.25_, S_0.5_, and S_0.75_ are 109.1 ± 4.2, 124.2 ± 4.4, and 130.6 ± 4.3 HV_0.2_, respectively. These values represent increases of 44.31%, 64.29%, and 72.75% compared to the hardness of S_0_ samples, which measured 75.6 ± 3.6 HV_0.2_. Notably, sample S_1_ exhibits the highest hardness at 135.0 ± 3.3 HV_0.2_, representing a remarkable increase of 78.57% compared to S_0_.

Room temperature tensile tests were performed on all samples to assess how the LaB_6_ content influences the mechanical characteristics of aluminum matrix composites. The findings, summarized in Table 2, are visually represented in Figure 8b.

This figure specifically highlights the impact of LaB_6_ content on the following key tensile parameters: the yield strength (YS) of the stress limit for the yield phenomenon of metals under external forces; the maximum stress values that metal materials can withstand during the tensile process, namely the fracture strength and ultimate tensile strength (UTS); and the elongation rate (A) of the deformation range that metal materials can withstand under tensile force. Notably, the LaB_6_ content emerges as a significant factor in determining each of these tensile attributes. It can be seen that, due to the significant forming issues, both the S_0_ and S_1_ samples exhibit numerous cracks, resulting in limited strength. With a further increase in LaB_6_ content to 0.25 wt.%, YS (125 ± 8 MPa), UTS (177 ± 3 MPa), and A (0.67 ± 0.26) increased by 115.52%, 205.17%, and 204.55%, respectively, compared with S_0_. However, among all alloy systems, the sample with 0.5 wt.% LaB_6_ (S_0.5_) demonstrates the best overall performance in terms of YS, UTS, and A. Nonetheless, its elongation is only 1.58%, indicating the persistence of certain challenges in the S_0.5_ samples, which is consistent with the EBSD detection results described earlier.

## 4. Discussion

### 4.1. Densification Behavior

This study has confirmed the effectiveness of LaB_6_ as a heterogeneous nucleating agent in the preparation of A2024 alloys using PBF-LB/M. The addition of 0.5 wt.% of LaB_6_ nanoparticles to high-strength aluminum alloys has been shown to decrease crack formation, improve machinability, and inhibit pore formation.

An excellent powder bed with spherical particles, good fluidity, and high powder quality was obtained by incorporating nano-LaB_6_ into the initial powder through an effective mechanical ball milling method. The presence of non-oxidized ceramics in the powder bed significantly alters the machinability of the A2024 alloy prepared using PBF-LB/M. This change is attributed to nano-LaB_6_’s high laser absorption rate and the dense powder bed’s ability to enhance the multiple reflection effect of the laser [40,41,42]. Together, these factors effectively address the processing challenges caused by the high reflectivity of aluminum alloy. The microstructure analysis of various samples reveals the presence of numerous pores and cracks within the A2024 aluminum alloy. These defects are primarily due to the inadequate melting of powder particles resulting from high reflectivity, insufficient liquid supply during solidification, and the poor flowability of low-viscosity melt.

The addition of 0.5 wt.% LaB_6_ nanoparticles to A2024 aluminum alloy has been found to enhance the machinability of the composite material. This improvement is attributed to the higher melt viscosity of the 0.5 wt.% LaB_6_/A2024 composite, which hinders Marangoni flow and minimizes spatter formation, resulting in improved formability. Despite the local molten pool temperature in the PBF-LB/M process, which exceeds the high melting point of LaB_6_ (2988 K), the analysis in Figure 4 indicates that no new substances similar to Al-La and Al-B are formed in the composite material. The unreacted LaB_6_ nanoparticles play a key role in increasing the overall viscosity of the molten pool, enabling the rapid filling of defects and thereby reducing pore formation and suppressing crack formation.

### 4.2. Grain Refinement by LaB_6_ Nanoparticles

**Figure 9:**
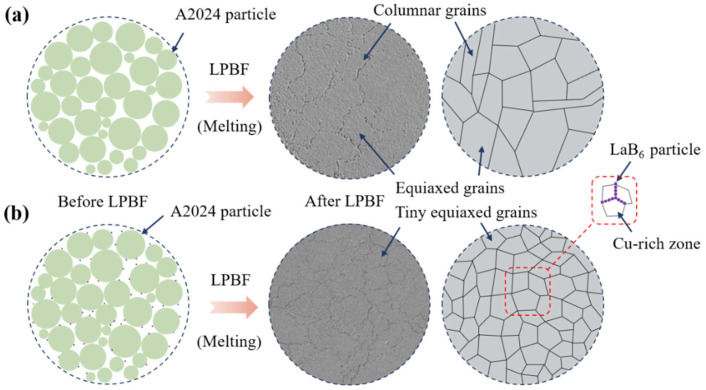
The formation mechanism and morphology of A2024 composite alloys during the PBF-LB/M process: (**a**) A2024 and (**b**) A2024 with LaB_6_ particle addition.

After the addition of nano-LaB_6_ particles, the grain sizes of the S_0_ sample and S_0.5_ composite are 10.3 μm and 9.0 μm, respectively (Figure 7a,d), representing a reduction of 12.6%. Nano-LaB_6_ particles demonstrate high stability and act as effective heterogeneous nucleating agents, increasing the nucleation probability during the alloy’s melting and solidification process. This promotes the formation of uniformly fine equiaxial grains from the original columnar grains in the PBF-LB/M component, leading to a decrease in grain size. Additionally, the coherent interface with a small lattice mismatch between LaB_6_ particles and α-Al facilitates their dissolution in the matrix melt, promoting the heterogeneous nucleation of Al [43]. Moreover, the significant Marangoni effect causes LaB_6_ particles to accumulate in the grain boundary region, hindering grain epitaxy growth. The combination of these mechanisms results in a significant reduction in the sample’s grain size (Figure 9) [44].

**Figure 10:**
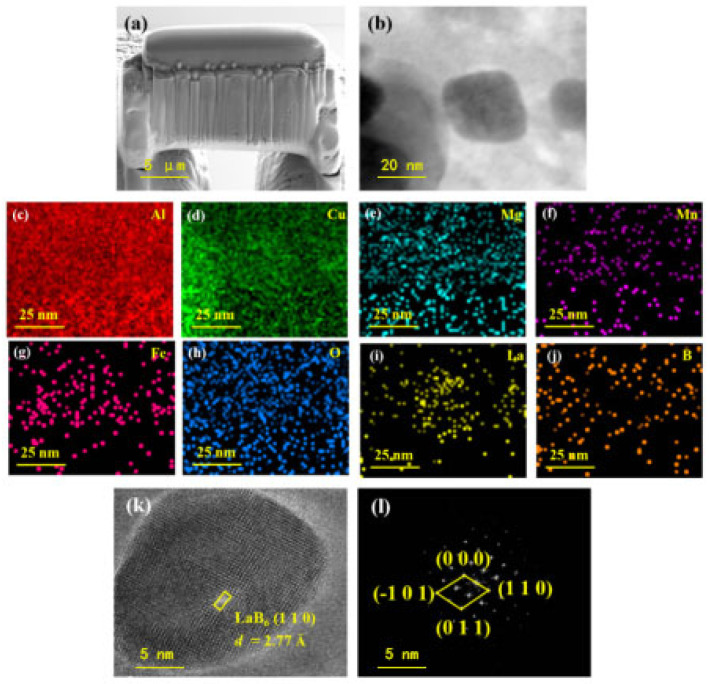
(**a**) Thinned TEM sample of LaB_6_/A2024 composite alloys via FIB technology; (**b**) TEM image of the LaB_6_/A2024 interface; (**c**–**j**) EDS mapping of Al, Cu, Mg, Mn, Fe, O, La, and B elements; (**k**,**l**) corresponding FFT and IFFT images.

To further investigate the incorporation of LaB_6_ into A2024 and examine the Cu-coated LaB_6_ interface, a sample was thinned using the focused ion beam (FIB) technique, as illustrated in Figure 10.

The diffraction pattern reveals that the outer white region corresponds to A2024 aluminum alloys, while the black area represents copper-coated nano-LaB_6_. Additionally, Figure 10 showcases the lattice transmission electron microscopy (TEM) and selected area electron diffraction (SAED) images of 0.5 wt.% LaB_6_/A2024 composites. In Figure 10k, the LaB_6_ unit cells are uniformly arranged without growth defects, with a measured unit cell distance of approximately 2.77 Å [45]. Moreover, large crystal cells attributed to Cu atoms are visible on the sample surface. The diffraction spots post-Fourier transform are depicted in Figure 10l, indicating the cubic nature of LaB_6_. The interplanar spacing of the lattice fringes measures 2.77 Å, corresponding to the (110) crystal plane spacing. These observations suggest robust interfacial adhesion between Cu and LaB_6_, resulting in the accumulation of LaB_6_ nanoparticles near Cu atoms at grain boundaries, which act as nucleation stabilizers through the Zener–pin effect and microstructural stabilizers. Furthermore, an increase in dislocation density at the LaB_6_–A2024 matrix interface enhances mechanical properties.

### 4.3. Mechanical Properties

The strengthening mechanisms of LaB_6_/A024 metal matrix composites (MMCs) prepared via PBF-LB/M can be categorized into four groups: fine-grain strengthening mechanism, load-transfer strengthening mechanism, dislocation strengthening mechanism, and Orowan strengthening mechanism, each operating independently. The yield strength is influenced by the combined contribution of all four mechanisms:

1. Fine-grain strengthening: The fine grains of PBF-LB/M-prepared A2024 composites are critical in determining their properties. The incorporation of LaB_6_ reinforcement particles provides extra nucleation points, aiding in the formation of the matrix phase and hindering grain growth. According to the Hall–Petch relation, materials exhibit increased strength as grain size decreases. Alloys with smaller grains generally have higher strength [45,46]. Moreover, the addition of reinforcement particles can enhance the ductility of metal matrix composites (MMCs). However, there is a limit to grain refinement, and an optimal amount of reinforcement particles is required for peak performance. This explains why the performance of S_0.75_ is not as good as that of S_0.5_. Furthermore, the high thermal conductivity of LaB_6_ particles speeds up heat dissipation in the molten pool, resulting in improved cooling rates and the formation of LaB_6_/A2024 composites:
  (1) $$∆\sigma_{HP}=k_{y}\left(d_{m}^{-1/2}-d_{0}^{-1/2}\right)$$
  where *d*_0_ is the grain size of the raw material, and *k_y_* is a constant related to the material.
2. Load-transfer strengthening occurs in PBF-LB/M-prepared LaB_6_/A2024 composites when external forces are applied, with nanoparticles at grain boundaries showing strong bonding properties. This facilitates load transfers and increases the composite’s strength [22,47]. Additionally, the application of loads improves the microhardness of the composite by preventing material plastic deformation due to dislocations between the matrix phase and LaB_6_ particles [23,48]. The strong interface bond between reinforced particles and the matrix promotes effective load transfers, ultimately enhancing the composite’s strength:
  (2) $$∆\sigma_{LT}=\frac{1}{2}v_{P}\sigma_{m}$$
  where *σ_m_* is the strength of the raw material.
3. Dislocation strengthening in composite materials is a result of mismatched thermal expansion and elastic modulus between the matrix and reinforcing particles. This mismatch becomes more significant at elevated temperatures, resulting in an increase in residual plastic strain and dislocation density. The improvement in the mechanical properties of composites is attributed to the dislocation density arising from the mismatch in thermal expansion coefficients. The relationship between dislocation density and the strength of composite materials can be described using the following formula [49,50,51]:
  (3) $$∆\sigma_{CTE}=\sqrt{3}\beta G_{m}b\sqrt{\frac{12v_{P}}{bd_{p}\left(1-v_{p}\right)}∆C∆T}$$
  The formula for dislocation strengthening includes a constant *β* of approximately 1.25; the shear modulus of the matrix *G_m_*; the Burger’s vector magnitude *b*; the particle content *v_p_*; the size of equiaxed particles *d_p_*; the difference in thermal expansion Δ*C*; and the temperature difference Δ*T* between ambient and processing temperatures.
4. When dislocations evade small strengthening particles, Orowan strengthening occurs. These particles serve as anchor points for dislocations, causing them to bend when subjected to external forces. This hinders the movement of dislocations and ultimately boosts the strength of metal matrix composites (MMCs) [51]. This process, known as Orowan strengthening, has been demonstrated in studies to be achievable only with small reinforcing particles (<1 μm) [49]. The transmission electron microscopy (TEM) images in this research on PBF-LB/M-fabricated LaB_6_/A2024 composites clearly display a uniform distribution of nanoscale LaB_6_ particles within the matrix, as depicted in Figure 10. Orowan strengthening plays a vital role in enhancing the strength of iron-based MMCs reinforced with particles and fabricated using the PBF-LB/M technique. This strengthening mechanism can be mathematically described using the following formula [51,52,53]:
  (4) $$\sigma_{Or}=\frac{0.4M}{\pi \sqrt{1-v}}\cdot \frac{G_{m}b}{L}\cdot ln\frac{\sqrt{2/3}d_{p}}{b}$$
  (5) $$L=\sqrt{\frac{2}{3}}d_{p}\left(\sqrt{\frac{\pi }{4v_{p}-1}}\right)$$
  *M*, *G_m_*, *b*, and *v* represent the Taylor factor, shear modulus of the matrix material, Burger’s vector, and Poisson’s ratio, respectively. *L* indicates the interparticle spacing, while *d_p_* and *v_p_* represent the size and volume fraction of the reinforced particles. This study shows that finely dispersed LaB_6_ particles effectively hinder the motion of dislocations, leading to an increase in the yield strength of A2024 aluminum-based composites.

## 5. Conclusions

In this study, we investigated the microstructure and mechanical properties of LaB_6_/A024 alloys produced using the PBF-LB/M process. The main findings are as follows:

1. In comparison to the unmodified A2024 alloy, the A2024 alloy modified with LaB_6_ exhibits an improvement in PBF-LB/M workability, leading to substantial reductions in porosity and solidification cracking.
2. Adding 0.5 wt.% LaB_6_ resulted in a transformation of the microstructure of the composite material into a single, uniform, equiaxed structure, with a refined grain size of 9.0 µm. Compared with the coarse and long columnar structure of traditional A2024 alloys, this uniform isomeric crystal structure significantly improves the solidification cracking resistance of the composite material. The improvements in this microstructure provide valuable insights into improving the overall performance and reliability of composite materials in practical applications.
3. The positive coating interaction between cubic ceramic nanoparticles and copper atoms (beneficial interface interaction between nanoparticles and matrix metal) highlights the significant efficacy of LaB_6_ as a non-uniform grain refiner for aluminum. A large portion of LaB_6_ nanoparticles is concentrated in the liquid phase during grain development, acting as microstructural stabilizers at grain boundaries through the Zener pinning mechanism.
4. The tensile strength of A2024 alloy is between 300 and 400 MPa, and the elongation is between 5 and 10%. However, these values may vary due to different preparation processes, heat treatment conditions, and alloy compositions. The tension strength and elongation at the frame of the A2024 alloy modified with 0.5 wt.% LaB_6_ are 251 ± 2 MPa and 1.58 ± 0.12%, respectively.

## Figures and Tables

**Table 1:** Elemental content of the commercial A2024 powder (wt.%).

Elements	Al	Cu	Mg	Mn	O	Si	Cr	Zn
Content	Balance	4.12	1.52	0.59	0.0189	0.011	<0.01	<0.01

**Table 2:** Mechanical properties of the PBF-LB/M as-built specimens.

Samples	YS (MPa)	UTS (MPa)	A (%)	HV_0.2_
S_0_	58 ± 2	58 ± 2	0.22 ± 0.09	75.6 ± 3.6
S_0.25_	125 ± 8	177 ± 3	0.67 ± 0.26	109.1 ± 4.2
S_0.5_	198 ± 7	251 ± 2	1.58 ± 0.12	124.2 ± 4.4
S_0.75_	287 ± 9	333 ± 6	0.72 ± 0.23	130.6 ± 4.3
S_1_	80 ± 6	84 ± 7	0.67 ± 0.26	135.0 ± 3.3

## Data Availability

The original contributions presented in the study are included in the article, further inquiries can be directed to the corresponding author.
